# The Role of Platelet Parameters in Predicting the Disease Severity of COVID-19 Patients: A Hospital-Based Study

**DOI:** 10.7759/cureus.51523

**Published:** 2024-01-02

**Authors:** Sampa Choudhury, Suparna Dubey, Mhasisielie Zumu, Mohammed Mustafa P P, Amrita Burma, Saurabh Kumar

**Affiliations:** 1 Department of Pathology, Andaman & Nicobar Islands Institute of Medical Sciences, Port Blair, IND; 2 Department of Pathology, School of Medical Sciences & Research, Sharda University, Greater Noida, IND; 3 Department of Medical Gastroenterology, The Madras Medical Mission Hospital, Chennai, IND; 4 Department of General Surgery, Andaman & Nicobar Islands Institute of Medical Sciences, Port Blair, IND; 5 Department of Community Medicine, Andaman & Nicobar Islands Institute of Medical Sciences, Port Blair, IND; 6 School of Medicine, Andaman & Nicobar Islands Institute of Medical Sciences, Port Blair, IND

**Keywords:** platelets, neutrophils, lymphocytes, hematology, covid-19

## Abstract

Background and objective

Globally, a large number of people have been impacted by the extremely contagious coronavirus disease 2019 (COVID-19) infection, and several pieces of literature were published on hematological parameter changes in this infection, particularly focusing on leucocytes. In this study, we have analyzed the platelet parameters and platelet-leucocyte ratios in COVID-19 patients and correlated them with the disease severity.

Methods

The COVID-19 patients who were hospitalized during the second peak of the pandemic were assessed. The platelet count and indices, platelet to lymphocyte ratio (PLR), neutrophil to platelet ratio (NPR), and clinical severity of each patient were analyzed. The significance of parameters and clinical severity was evaluated using analysis of variance (ANOVA) and the Kruskal Wallis test. A bivariate analysis was performed to assess the strength of the association. Receiver operating characteristic (ROC) curves were plotted to detect the predictive value of parameters for disease severity.

Results

The data of 132 COVID-19 patients has been evaluated. The average mean age was 45.60 ± 15.76 years with slight female predominance. Thrombocytopenia was found in 33% of cases with the majority of them being mild. Age, platelet distribution width (PDW), and PLR and NPR parameters were significantly (p-value <0.05) associated with disease severity. ROC curves showed age, PDW, PLR, NPR, absolute neutrophil count (ANC), and absolute lymphocyte count (ALC) had significant prediction values for clinical severity.

Conclusions

Platelet parameters may not accurately reflect the severity of the disease, but when combined with leucocyte parameters and their ratios (PLR and NPR), they offer important information about disease severity.

## Introduction

Coronavirus disease 2019 is a highly infectious airborne disease caused by the severe acute respiratory syndrome coronavirus 2 (SARS-CoV-2) virus, which was declared a pandemic on 11 March 2020 by the World Health Organisation (WHO), and to date, millions of people have been infected with many dying all over the world due to this deadly infection [1,2]. SARS-CoV-2 is an RNA virus that is also known as coronavirus because of its ultrastructural club-shaped spikes resembling solar corona on electron microscopy [3]. The virus has undergone numerous mutations since its first outbreak in the early months of 2020, giving rise to multiple variants. Some of the variants, like Alpha (B.1.1.7), Beta (B.1.351), Gamma (P.1), Delta (B.1.671.2), and Omicron (B.1.1.529) are identified by the WHO as variants of concern (VOCs). Among them, the Delta variant, which is responsible for the catastrophic second wave in India, has mutated spike protein with enhanced angiotensin-converting enzyme-2 (ACE-2) binding capacity, making it highly infectious and fatal [4,5]. After evasion into the host cells, the virus, irrespective of any variants, induces a series of events, especially renin-angiotensin-aldosterone axis derangement, immune dysregulation, and cytokine storm leading to tissue injury and destruction [6]. The JN.1 variant, with an additional mutation in spike protein and high transmissibility, was recently classified as a distinct variant of interest (VOI) from the parent lineage of BA.2.86 by WHO. This variant has been identified in a few states of India with rising concern, but its pathogenesis and clinical behavior are yet to be explored in detail [7].

The clinical features of COVID-19 range from asymptomatic to fatal pneumonia, with the most common being influenza-like symptoms such as fever, dry cough, and weakness. In severe cases, patients may present with acute respiratory distress syndrome (ARDS), metabolic acidosis, septic shock, and multi-organ failure [3,8]. Changes in the hematological parameters, both quantitative and morphological, have been widely investigated in the recent past. The most commonly observed changes are lymphopenia, neutrophilia, elevated neutrophil-to-lymphocyte ratio (NLR), thrombocytopenia, elevated D-dimer, and prothrombin time. These changes become more evident with the severity of the disease. The morphological changes of blood cell components include immature granulocytes, toxic granulation, pseudo-Pelger Huet deformity, atypical lymphocytes, activated monocytes, hyperchromatic platelets, and platelet clumps [6,9]. However, changes in platelets and their indices related to this highly contagious viral infection are less explored in the research articles.

Platelets, the anucleate blood component, are crucial for the coagulation pathway as well as the immune system. The antimicrobial defense mechanism of platelets, comprising both innate and adaptive immunity, is well known to protect against many bacterial, viral, and parasitic infestations [10]. Hence, we aim to study platelet parameter changes in COVID-19 patients and their relationship with disease severity. This study also emphasizes the value of a simple, reasonably priced complete hemogram that provides a wealth of information to aid in patient care in settings with limited resources.

## Materials and methods

Study design and participants

This is a cross-sectional retrospective study performed in the Department of Pathology of a tertiary care hospital on a tropical Indian island. Sampling was done by the convenience sampling method. All complete hemogram reports of COVID-19-positive patients on their admission day from April 2021 to July 2021, the second peak of the COVID pandemic, irrespective of age and gender, were selected. Only patients with positive real-time polymerase chain reaction (RT-PCR) tests were included.

Data collection

A data collection format was used to assess age, gender, clinical severity and various platelets’ parameters like platelet count (normal 150-400 x10^9^/L), mean platelet volume (MPV: 8.6-15.5 fL), platelet distribution width (PDW: 8.3-25 fL), plateletcrit (PCT: 0.22-0.24 %), platelet large cell ratio (P-LCR: 15-35 %), platelet to lymphocyte ratio (PLR), and neutrophil to platelet ratio (NPR). The absolute lymphocyte count (ALC) and absolute neutrophil count (ANC) were also calculated for the evaluation of PLR and NPR. All samples were run by the hematology analyzer machine Transasia Sysmex XN 1000 (Sysmex, Mumbai, India) and registered in a hematology register. Data were also collected from patients’ medical records for clinical categorization of the disease.

Definitions

Platelet count <150x10^9^/L was considered thrombocytopenia, whereas a count >400x10^9^/L was considered thrombocytosis. Thrombocytopenia was graded as a mild or moderate category if the count was ≥ 100 to <150x10^9^/L; 50 to <100x10^9^/L respectively.

The clinical severity of the COVID-19-positive patients was categorized according to the Indian Council of Medical Research (ICMR) guidelines. The mild disease is characterized by upper respiratory tract symptoms and/or fever without shortness of breath or hypoxia while the moderate category has either a respiratory rate ≥ 24/min, breathlessness, or oxygen saturation (Spo2) of 90% to ≤ 93% on room air. The severe disease is reserved for patients with a respiratory rate >30/min, breathlessness, or Spo2 <90% on room air [11].

Analysis plan

Data were entered in Microsoft Excel 2013 (Microsoft Corporation, Redmond, WA, USA) and statistical analysis was done using SPSS^©^ IBM trial software version 26.0 (IBM Corp., Armonk, NY). The descriptive data were presented as mean, median, frequencies, proportions, and standard deviation. The normality distribution was assessed by the Shapiro-Wilk test.

The significance of platelet parameters and the clinical severity (mild, moderate, and severe) of the COVID-19 disease was evaluated by using analysis of variance (ANOVA) for parametric data and the Kruskal-Wallis test for non-parametric data. Tukey HSD (Honestly Significant Difference) post-hoc test was conducted for parameters showing a significant p-value in ANOVA.

Bivariate analysis was performed to generate the odds ratio (OR) for the strength of association of various parameters, which showed deviations from normal ranges, with the clinical severity. For this purpose, 130 adult cases were divided into two groups, severe (moderate and severe disease) and non-severe group (mild disease), excluding two children. The parameters with known normal ranges for adults are divided into normal and abnormal groups. Fisher’s exact test was done for significance. 95% confidence interval for OR, and p-value less than 0.05 were used for the significance of the test.

To establish the predictive value of each parameter for severe disease, receiver operator characteristic (ROC) curves were plotted. An area under the curve (AUC) of ≥ 0.65 was considered acceptable.

## Results

A total of 132 COVID-19-positive cases were evaluated during this time period. The age ranges from 1 to 80 years with a mean age of 45.60 ± 15.76 years with slight female predominance (Male: Female ratio-0.9:1.0). In the clinical severity, the most common was the mild category (49.2%), followed by almost equal percentages of the moderate (25.8%) and severe categories (25%). There were 43 cases (33%) of thrombocytopenia and only 4 cases (3%) of thrombocytosis. Among the thrombocytopenia cases, 27 (63%) and 16 (37%) cases were in the mild and moderate grades, respectively.

Clinical severity worsened with the increasing age of the patient, and significant ‘p’ values were observed between the mild to moderate and mild to severe categories (Table 1).

**Table 1:** One-way ANOVA test and post-hoc for age ANOVA, analysis of variance

Variables	Clinical severity			P-value (ANOVA)
	Mild	Moderate	Severe	
Age (Mean ± Standard deviation)	38.63±15.122	49.74±11.927	55.06±13.872	0.0001
	Clinical severity (I)	Clinical severity (J)	Mean difference (I-J)	P value (Post-hoc)
	Mild	Moderate	-11.105	0.001
		Severe	-16.430	0.000
	Moderate	Mild	11.105	0.001
		Severe	-5.325	0.271
	Severe	Mild	16.430	0.000
		Moderate	5.325	0.271

Among the platelet parameters, PDW, PLR, and NPR showed an increase in median values with worsening of the disease condition and a significant (p<0.05) association with disease severity. Other parameters depicted insignificant values (Table 2).

**Table 2:** Correlation of platelet parameters with clinical severity *Kruskal-Wallis test; #One-way ANOVA test IQR, interquartile range; MPV, mean platelet volume; NPR, neutrophil to platelet ratio; PCT, plateletcrit; PDW, platelet distribution width; P-LCR, platelet large cell ratio; PLR, platelet to lymphocyte ratio; SD, standard deviation

Variables	Unit	Clinical severity (Median (Interquartile range))			P value
		Mild	Moderate	Severe	
Platelet count (10^9^/L)	Median (IQR)	175 (87)	156 (148.25)	174 (133.5)	0.635^*^
MPV (fL)	Mean ± SD	11.629 ± 2.007	11.944 ± 1.868	12.033 ± 1.708	0.547^#^
PDW (fL)	Median (IQR)	16.10 (0.90)	16.35 (0.925)	16.0 (1.1)	0.014^*^
PCT (%)	Median (IQR)	0.20 (0.09)	0.19 (0.156)	0.19 (0.1)	0.772^*^
P-LCR (%)	Mean ± SD	38.529 ± 13.521	41.282 ± 13.419	41.597 ± 11.257	0.437^#^
PLR	Median (IQR)	123 (133.5)	159.5 (186.25)	247 (232)	0.001^*^
NPR	Median (IQR)	26 (18)	37.5 (34.25)	63 (76.5)	0.0001^*^

In bivariate analysis, no platelet parameters showed a strong association with severe disease. However, neutrophilia (OD-6.57) and lymphopenia (OD-1.96) were associated with clinical severity (Table 3).

**Table 3:** Bivariate analysis of various parameters with clinical severity ALC, absolute lymphocyte count; ANC, absolute neutrophil count; MPV, mean platelet volume; PCT, plateletcrit; P-LCR, platelet large cell ratio

Variables	Categories	Clinical severity		Total (n=130)	P-value (Fisher’s exact test)	Odds ratio (95% CI)
		Severe (n=33)	Non-severe (n=97)			
Platelet count	Thrombocytopenia	12	31	43	0.898	1.22(0.53-2.81)
	Normal	20	63	83		1
	Thrombocytosis	1	3	4		1.05(0.1-10.67)
MPV	Low	0	4	4	0.280	—
	Normal	31	89	120		1
	High	2	4	6		1.44(0.25-8.23)
PCT	Low	19	48	67	0.559	1.7(0.64-4.52)
	Normal	7	30	37		1
	High	7	19	26		1.58(0.48-5.22)
P-LCR	Low	0	2	2	0.518	—
	Normal	11	35	46		1
	High	22	60	82		1.17(0.51-2.69)
ALC	Lymphopenia	21	43	64	0.125	1.96(0.82-4.65)
	Normal	12	54	66		1
ANC	Neutropenia	0	7	7	0.0001	—
	Normal	8	61	69		1
	Neutrophilia	25	29	54		6.57(2.64-16.34)

**Figure 1:**
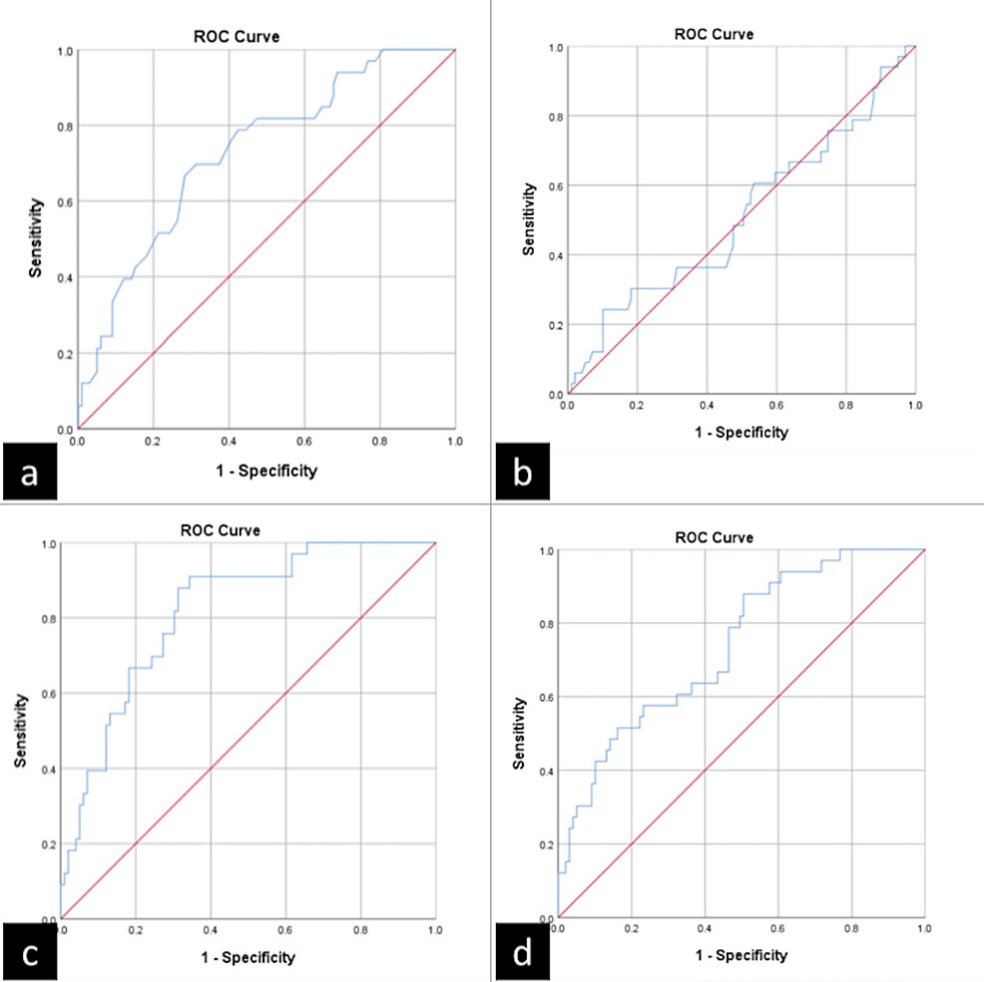
ROC curves of a) Age, b) Platelet count, c) ANC, and d) ALC ALC, absolute lymphocyte count; ANC, absolute neutrophil count; ROC, receiver operating characteristic

**Figure 2:**
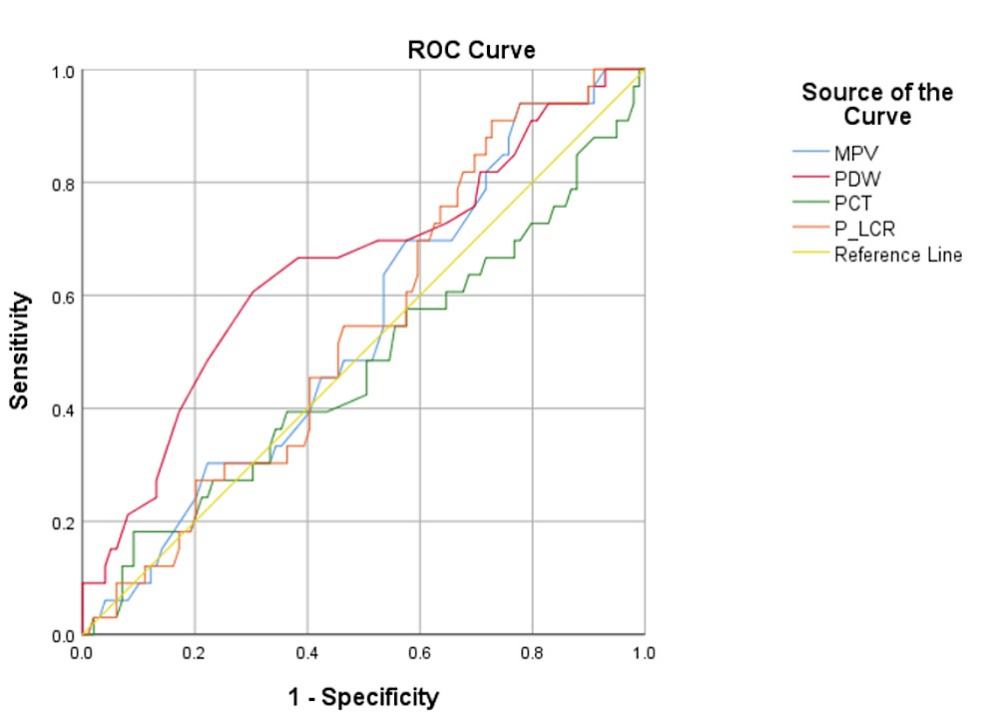
ROC curve of platelet indices MPV, mean platelet volume; PCT, plateletcrit; PDW, platelet distribution width; P-LCR, platelet large cell ratio; ROC, receiver operating characteristic

**Figure 3:**
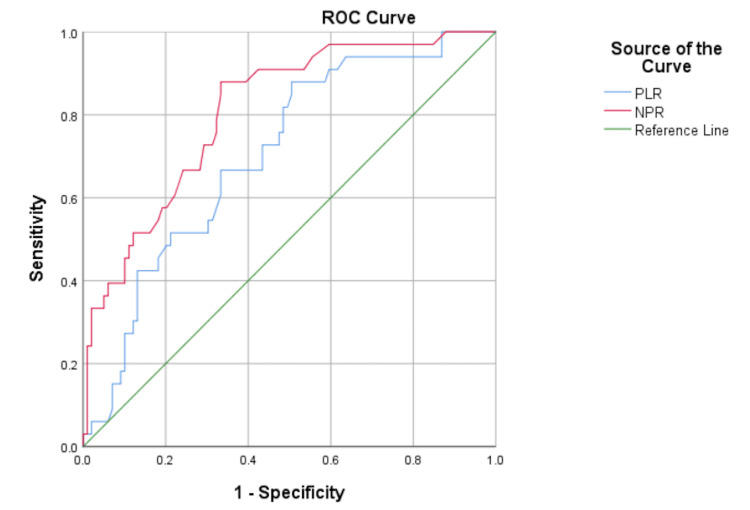
ROC curve of PLR and NPR NPR, neutrophil to platelet ratio; PLR, platelet to lymphocyte ratio; ROC, receiver operating characteristic

The ROC curve analysis (Figures 1-3) for predicting risk factors for severe COVID-19 patients showed significant ‘p’ values with ≥ 0.65 AUC in age, PDW, PLR, NPR, ANC, and ALC. The AUC of NPR (0.80) and ANC (0.81) were larger than the rest of the other parameters. The optimal cutoff values, sensitivity, and specificity are depicted in Table 4.

**Table 4:** AUC and cutoffs of different parameters ALC, absolute lymphocyte count; ANC, absolute neutrophil count; AUC, area under the ROC curve; MPV, mean platelet volume; NPR, neutrophil to platelet ratio; PCT, plateletcrit; PDW, platelet distribution width; P-LCR, platelet large cell ratio; PLR, platelet to lymphocyte ratio

Parameters	AUC	95% CI	P-value	Optimal cutoff	Sensitivity (%)	Specificity (%)
Age (years)	0.72	0.63-0.82	0.000	>43.5	81.8	52.5
Platelet count (10^9^/L)	0.48	0.36-0.60	0.827	—	—	—
MPV (fL)	0.54	0.43-0.65	0.473	—	—	—
PDW (fL)	0.65	0.53-0.76	0.010	>15.75	81.8	29.3
PCT (%)	0.47	0.35-0.59	0.651	—	—	—
P-LCR (%)	0.54	0.43-0.65	0.446	—	—	—
PLR	0.70	0.60-0.80	0.000	>131.5	84.8	49.5
NPR	0.80	0.72-0.89	0.000	>36.5	87.9	66.7
ANC	0.81	0.74-0.89	0.000	>6047.5	90.9	65.7
ALC	0.73	0.64-0.83	0.000	<1231	87.9	48.5

## Discussion

The SARS-CoV-2 and its mutated virulent variants lead to multiple pandemic waves worldwide with a significant burden of morbidity and mortality. In our study, people affected during the second wave of the pandemic in this remote Indian island were analyzed. The mean age of the COVID patients was 45.60 ± 15.76 years with a younger (38.63 ± 15.12 years) population in the mild category and the elderly (55.06 ± 13.87 years) in the severe category. The finding is comparable with other studies [12,13]. The higher prevalence of associated comorbidities in the elderly may be the reason why disease severity rises with age. The majority of Indian studies found a male predominance with plausible explanations, including smoking habits, outdoor activities, and gender differences, in India [12-14]. However, we observed slight female predominance in this geographical region.

We have observed 33% thrombocytopenia in hospital-admitted patients, but interestingly, we couldn’t find any correlation between a low platelet count and clinical severity. Some authors, like Güçlü et al. [1] and Arise et al. [15], reported 25% and 23.9% cases of thrombocytopenia, respectively. Moreover, both studies mentioned the non-correlation of thrombocytopenia with disease severity. These findings are quite similar to our findings. However, other authors like Lippi et al. [16] and Li et al. [17] opined that low platelet count is significantly associated with severe disease and mortality. A review article by Wool et al. [18] stated that COVID-19-induced thrombocytopenia is characteristically mild, which is comparable with our findings. Several mechanisms may contribute to the low platelet count in COVID-19 infection. This could be cytokine storm-induced bone marrow suppression, excessive consumption during microthrombi formation, or due to splenic or hepatic sequestration [9,18]. Isolated virus-induced immune thrombocytopenia cases were also reported due to the presence of anti-platelet antibodies or lupus anticoagulant antibodies [19,20].

Research on platelet indices varied widely, with some writers focusing solely on MPV and PDW while others examined all indices. Some have compared the results between survivors and non-survivors while others have compared them with severe or not-severe categories. In our study, we have included all parameters on admission day and only PDW is found to be significantly correlated with disease severity (p<0.05). We didn’t include the follow-up cases. A research article by Ozcelik et al. [21] observed significantly lower MPV and higher PDW in COVID-19 patients compared to the influenza infection. Another article mentioned significantly low PCT and high MPV, PDW, and P-LCR in COVID-19 patients compared to controls [22]. A study conducted by Asrie et al. [15] at a northwest Ethiopian hospital concluded that increased PDW is significantly associated with disease severity. The freshly released immature, large platelets that were released as compensation for platelet destruction may have increased the PDW. However, they couldn’t find any obvious association with MPV, PCT, or P-LCR. On the contrary, Ravindra et al. [23] claimed that there was no discernible difference between survivor and non-survivor groups, as well as between moderate and severe categories, with respect to MPV and PDW. Another study by Çavus et al. [24] reported a significantly lower PCT in COVID-19 patients compared to controls; however, there is no difference between severe versus mild categories.

The PLR, an easily available novel inflammatory marker in most clinical settings, has widely been used as a prognostic marker for neoplastic as well as cardiovascular diseases in the pre-COVID era [25]. Recently, a meta-analysis on PLR in COVID-19 infection opined that elevated PLR is associated with increased morbidity and mortality [26]. We also observed similar findings with gradually increasing values of PLR from mild, moderate, to severe disease, which is statistically significant (p-value 0.001). The elevated PLR ratio in critically ill patients is due to the marked lymphopenia compared to the thrombocytopenia. Directly virus-induced pyroptosis, enhanced utilization by interleukin-6 (IL-6), and acute tissue sequestration seem to be the mechanisms of lymphopenia [26].

Another inflammatory marker, NPR, is found to be associated with cardiovascular and cerebrovascular events like infective endocarditis, myocardial infarction, hemorrhagic transformation of acute ischemic stroke, and hematoma expansion in spontaneous intracerebral hemorrhage [27-30]. We have first analyzed this marker in relation to COVID-19 infection. Our result revealed that the increasing value of NPR is highly associated with disease severity. The relationship of NPR with disease severity could be the reflection of neutrophilia more than thrombocytopenia. The virus-induced inflammation or a subsequent bacterial infection may both contribute to neutrophilia in SARS-CoV-2 infection [9].

For the evaluation of PLR and NPR, we have also calculated ALC and ANC, respectively, and correlated with disease severity. Our findings showed lymphopenia and neutrophilia on admission day were highly associated with disease severity. A meta-analysis by Henry et al. [31] also opined similar findings.

In this present study, ROC curve analysis revealed high sensitivity ranges from 81.8% to 90.9% in age, PDW, PLR, NPR, ANC, and ALC parameters. However, specificity was low in all parameters. PLR with a cut-off of more than 131.5 has 84.8% sensitivity and 49.5% specificity, which is comparable with the findings of Ravindra et al. [23]. On the other hand, Wang et al. [32] observed 77.42% sensitivity and 75% specificity with an optimal cut-off of 189.11 of PLR. Additionally, the comparison of other parameters is restricted by the dearth of studies using ROC curve analysis.

Limitations

There are a few caveats in our study. First, we took the parameters only on admission day. The parameters on follow-up days were not assessed, which lacks information about the changes related to any improvement or worsened condition of the disease. Second, we did not have clinical details and therefore, could not exclude patients on antiplatelet therapy, or with hematological diseases, cancer, or pregnancy, which can affect the platelet parameters.

## Conclusions

We attempted to evaluate platelet parameters in COVID-19 patients and have noted that platelet parameters by themselves may not accurately and independently reflect the severity of the disease, but when combined with leucocyte parameters (ALC and ANC) and their ratios (PLR and NPR), they will offer important information about disease severity. Additionally, without performing a lot of expensive serological (D-dimer assay, C-reactive protein, or IL-6) and radiological (high-resolution computed tomography of the chest) investigations, these parameters can be easily obtained from a complete hemogram, which is accessible for populations of all income levels.

## References

[1] Güçlü E, Kocayiğit H, Okan HD (2020). Effect of COVID-19 on platelet count and its indices. Rev Assoc Med Bras (1992).

[2] Yun H, Sun Z, Wu J, Tang A, Hu M, Xiang Z (2020). Laboratory data analysis of novel coronavirus (COVID-19) screening in 2510 patients. Clin Chim Acta.

[3] Patel KA, Parmar AR, Sharma BS, Thacker M, Patel N, Patel B (2021). A study of hematological parameters in patients with COVID-19 infection at a tertiary care centre. J Pathol Nep.

[4] Aleem A, Akbar Samad AB, Vaqar S (2022). Emerging Variants of SARS-CoV-2 and Novel Therapeutics Against Coronavirus (COVID-19). https://www.ncbi.nlm.nih.gov/books/NBK570580/?utm_medium=email&utm_source=transaction.

[5] Kunal S, Aditi Aditi, Gupta K, Ish P (2021). COVID-19 variants in India: potential role in second wave and impact on vaccination. Heart Lung.

[6] Kaur G, Sandeep F, Olayinka O, Gupta G (2021). Morphologic changes in circulating blood cells of COVID-19 patients. Cureus.

[7] Perappadan BS (2023). Government issues COVID-19 alert, says no clustering of JN.1 cases. New Delhi December 20.

[8] Zhou F, Yu T, Du R (2020). Clinical course and risk factors for mortality of adult inpatients with COVID-19 in Wuhan, China: a retrospective cohort study. Lancet.

[9] Rahman A, Niloofa R, Jayarajah U, De Mel S, Abeysuriya V, Seneviratne SL (2021). Hematological abnormalities in COVID- 19: a narrative review. Am J Trop Med Hyg.

[10] Ali RA, Wuescher LM, Worth RG (2015). Platelets: essential components of the immune system. Curr Trends Immunol.

[11] (2023). Ministry of Health and Family Welfare, Government of India. Clinical guidance for management of adult COVID‐19 patients. https://cdnbbsr.s3waas.gov.in/s3850af92f8d9903e7a4e0559a98ecc857/uploads/2023/04/2023040555.pdf.

[12] Ahmad S, Kumar P, Shekhar S, Saha R, Ranjan A, Pandey S (2021). Epidemiological, clinical, and laboratory predictors of in-hospital mortality among COVID-19 patients admitted in a tertiary COVID dedicated hospital, northern India: a retrospective observational study. J Prim Care Community Health.

[13] Chauhan NK, Shadrach BJ, Garg MK (2021). Predictors of clinical outcomes in adult COVID-19 patients admitted to a tertiary care hospital in India: an analytical cross-sectional study. Acta Biomed.

[14] Elavarasi A, Raju Sagiraju HK, Garg RK (2022). Clinical features, demography, and predictors of outcomes of SARS-CoV-2 infection at a tertiary care hospital in India: a cohort study. Lung India.

[15] Asrie F, Tekle E, Gelaw Y (2022). Baseline thrombocytopenia and disease severity among COVID-19 patients, Tibebe Ghion specialized hospital COVID-19 treatment center, northwest Ethiopia. J Blood Med.

[16] Lippi G, Plebani M, Henry BM (2020). Thrombocytopenia is associated with severe coronavirus disease 2019 (COVID-19) infections: a meta-analysis. Clin Chim Acta.

[17] Li Q, Cao Y, Chen L (2020). Hematological features of persons with COVID-19. Leukemia.

[18] Wool GD, Miller JL (2021). The impact of COVID-19 disease on platelets and coagulation. Pathobiology.

[19] Menakuru SR, Priscu A, Dhillon VS, Salih A (2022). The development of immune thrombocytopenia due to COVID-19 presenting as menorrhagia. Cureus.

[20] Humbert S, Razanamahery J, Payet-Revest C, Bouiller K, Chirouze C (2020). COVID-19 as a cause of immune thrombocytopenia. Med Mal Infect.

[21] Ozcelik N, Ozyurt S, Yilmaz Kara B, Gumus A, Sahin U (2021). The value of the platelet count and platelet indices in differentiation of COVID-19 and influenza pneumonia. J Med Virol.

[22] Shankaralingappa A, Tummidi S, Arun Babu T (2022). Diagnostic value of platelet indices in COVID 19 infection: a case-control study from a single tertiary care center. Egypt J Intern Med.

[23] Ravindra R, Ramamurthy P, Aslam S SM, Kulkarni A, K S, Ramamurthy PS (2022). Platelet indices and platelet to lymphocyte ratio (PLR) as markers for predicting COVID-19 infection severity. Cureus.

[24] Çavus Z, Tezdönen M, Çekme M, Türkmen ÜA (2021). Determination of plateletcrit, mean platelet volume in patients with COVID-19 pneumonia. J Immunol Clin Microbiol.

[25] Kurtul A, Ornek E (2019). Platelet to lymphocyte ratio in cardiovascular diseases: a systematic review. Angiology.

[26] Sarkar S, Kannan S, Khanna P, Singh AK (2022). Role of platelet-to-lymphocyte count ratio (PLR), as a prognostic indicator in COVID-19: a systematic review and meta-analysis. J Med Virol.

[27] Wei XB, Liu YH, He PC, Yu DQ, Tan N, Zhou YL, Chen JY (2017). The impact of admission neutrophil-to-platelet ratio on in-hospital and long-term mortality in patients with infective endocarditis. Clin Chem Lab Med.

[28] Somaschini A, Cornara S, Demarchi A (2020). Neutrophil to platelet ratio: a novel prognostic biomarker in ST-elevation myocardial infarction patients undergoing primary percutaneous coronary intervention. Eur J Prev Cardiol.

[29] He W, Ruan Y, Yuan C (2019). High neutrophil-to-platelet ratio is associated with hemorrhagic transformation in patients with acute ischemic stroke. Front Neurol.

[30] Li Y, Yang X, Zhou H, Hui X, Li H, Zheng J (2023). A high neutrophil-to-platelet ratio is associated with hematoma expansion in patients with spontaneous intracerebral hemorrhage: a retrospective study. BMC Neurol.

[31] Henry B, Cheruiyot I, Vikse J (2020). Lymphopenia and neutrophilia at admission predicts severity and mortality in patients with COVID-19: a meta-analysis. Acta Biomed.

[32] Wang W, Zhao Z, Liu X (2020). Clinical features and potential risk factors for discerning the critical cases and predicting the outcome of patients with COVID-19. J Clin Lab Anal.

